# Exploring molecular mechanisms of radioactive iodine therapy in thyroid cancer using single-cell RNA sequencing data

**DOI:** 10.1007/s12672-025-04317-x

**Published:** 2026-01-03

**Authors:** Lin Guo, Yiren Feng, Senhe Jiang, Xuehan Wang, Gang Jin

**Affiliations:** 1 Department of Nuclear Medicine, The Second Affiliated Hospital of Harbin Medical University, Harbin, 150000 China; 2 Department of Otolaryngology, The First Affiliated Hospital of Harbin Medical University, Harbin, 150000 China

**Keywords:** THCA, URDEGs, Pathways, Prognostic risk model

## Abstract

**Objective:**

To investigate the role of ubiquitination-related differentially expressed genes (URDEGs) in thyroid carcinoma (THCA) and their implications for predicting responses to radioactive iodine (RAI) therapy.

**Methods:**

We analyzed data from The Cancer Genome Atlas (TCGA) and the Gene Expression Omnibus (GEO) databases using TCGA biolinks and GEO query tools for data acquisition and normalization. Differential expression analysis was performed with DESeq2 to identify URDEGs between RAI-sensitive and RAI- refractory groups. Gene Ontology (GO) and Kyoto Encyclopedia of Genes and Genomes (KEGG) enrichment analyses were conducted using ClusterProfiler. A prognostic risk model was built using Cox regression and Least Absolute Shrinkage and Selection Operator (LASSO) regression. Single-cell RNA sequencing data were analyzed with Seurat for clustering and annotation. Gene Set Variation Analysis (GSVA) was used to assess pathway enrichment across various cell subpopulations.

**Results:**

A total of 25 URDEGs were identified, which were enriched in processes related to cytoskeleton organization and pathways including the peroxisome proliferator-activated receptor (PPAR) signaling pathway. A prognostic model based on four URDEGs—INSM2, TAGLN3, MDGA2, and SIK1—demonstrated strong predictive capability. Immune infiltration analysis revealed significant differences in immune cell composition between high- and low-risk groups. Single-cell analysis identified 74,497 cells across 22 clusters and nine cell types, revealing gene expression patterns that highlight cellular heterogeneity.

**Conclusion:**

URDEGs show potential as biomarkers for predicting RAI therapy response and prognosis in THCA. These findings provide insights for personalized therapeutic strategies targeting specific molecular pathways in THCA patients.

**Supplementary Information:**

The online version contains supplementary material available at 10.1007/s12672-025-04317-x.

## Introduction

Thyroid cancer, known to be a common endocrine malignancy, has seen a rising incidence in recent years. In 2020, around 220,000 new cases were reported in China, representing a substantial portion of the global total. Patients are typically presented with painless thyroid nodules, while some may also exhibit lymphadenopathy. Although most cases are classified as low risk, a subset of patients may progress to more aggressive subtypes or develop distant metastases. Recent studies have highlighted the heterogeneity of thyroid cancer and its association with genetic mutations, identifying particular genes essential for the onset and advancement of the disease is crucial [1].

Despite the expanding options for early diagnosis and treatment, a research gap persists in the management of radioactive iodine (RAI)-refractory thyroid cancer. The effectiveness and tolerability of current treatment modalities, including surgery, RAI therapy, and targeted drug therapy, are known to vary among patients. Hence, it is crucial to conduct research targeting this particular patient cohort to advance the development of more efficacious treatment approaches.

Immune checkpoint inhibitors (ICIs) have fundamentally transformed cancer treatment by markedly augmenting the capacity of the immune system to identify and eliminate cancerous cells. These inhibitors, such as PD-1, PD-L1, and CTLA-4 blockers, have shown considerable efficacy in treating different types of malignancies, resulting in enhanced survival rates for patients with advanced cancers [2]. In spite of these advancements, the clinical utilization of ICIs continues to pose challenges, primarily due to the onset of immune-related adverse events (irAEs) and the heterogeneity in patient responses. Consequently, there is an immediate necessity to investigate innovative therapeutic approaches to bolster efficacy while minimizing toxic effects [3].

Traditional cancer treatment methods, such as chemotherapy and radiation, often lack specificity and could cause significant collateral damage to healthy tissues. These treatments also fail to address the underlying immune evasion mechanisms employed by tumors. In contrast, ICIs specifically target immune checkpoints, which are regulatory pathways in T cells that tumors exploit to avoid immune detection. However, the efficacy of ICIs is often limited by intrinsic and acquired resistance mechanisms, highlighting the need for novel approaches to overcome these barriers [4]. This study employs advanced bioinformatics tools and single-cell RNA sequencing to identify URDEGs associated with RAI therapy sensitivity and resistance in THCA, offering a more precise and potentially effective therapeutic strategy [5].

Ubiquitination serves as a pivotal post-translational modification, governing protein turnover, signaling cascades, and numerous other cellular functions. Its role in cancer progression and immune homeostasis has been well-documented. Prior studies have indicated that perturbations within ubiquitination pathways may underlie immune evasion in a spectrum of cancers, notably THCA [6]. For example, variations in the expression levels of ubiquitin ligases and deubiquitinating enzymes have been associated with the regulation of immune checkpoint molecules and the tumor microenvironment, ultimately impacting the efficacy of ICIs [7]. Furthermore, single-cell RNA sequencing has revealed novel insights into the heterogeneity of cells and the dynamic alterations occurring within the tumor microenvironment. This technique has facilitated the identification of specific cell populations and the pathways associated with immune resistance [8]. Distinct from traditional bulk RNA sequencing, which merely captures the average gene expression profile across a heterogeneous cell mixture, single-cell RNA sequencing offers the capability to examine gene expression at the resolution of individual cells. This methodology excels in revealing the complex makeup of the tumor microenvironment, which encompasses diverse cell types such as cancer cells, immune cells, and stromal elements. By differentiating these diverse cell populations, single-cell RNA sequencing not only elucidates their unique contributions to tumor progression and immune escape but also monitors their temporal dynamics or alterations in response to therapeutic interventions. Such a granular level of resolution is indispensable for pinpointing therapeutic targets that might otherwise escape detection using standard methodologies.

This study aims to clarify the molecular mechanisms governing RAI therapy sensitivity and resistance in THCA, emphasizing URGs. We analyzed TCGA and GEO database data using differential expression, GO, and KEGG enrichment analyses, along with single-cell RNA sequencing, to identify and describe these genes. By constructing a prognostic risk model based on URDGEs, we aim to provide a robust predictive tool for clinical outcomes and potential therapeutic targets for overcoming resistance to RAI therapy in THCA.

In conclusion, our research employs advanced bioinformatics and single-cell technologies to overcome the shortcomings of conventional cancer therapies and ICIs. By investigating URDGEs and their involvement in therapeutic resistance, we strive to augment existing treatments and facilitate the development of innovative therapeutic approaches for THCA. This technique significantly enhances our understanding of the molecular mechanisms underlying cancer resistance, while simultaneously presenting opportunities to improve patient outcomes through personalized treatment strategies.

## Materials and methods

### Data download

We used The R package TCGAbiolinks [9] (Version 2.30.0) from (The Cancer Genome Atlas, TCGA) database (https://portal.gdc.cancer.gov/) to download thyroid cancer (THCA) data set (TCGA-THCA) and acts as a validation set was examined, and eliminate the lack of clinical information of data samples, following the removal of data entries lacking complete clinical details, 240 samples of Radioactive Iodine Sensitivity Papillary Thyroid Cancer (RAI Sensitivity PTC) with clinical information and 34 samples of Radioactive Iodine Resistance Papillary Thyroid Cancer (RAI Resistance PTC) with clinical information were collected in a Counts format. Simultaneously, the data were standardized to the FPKM (Fragments Per Kilobase per Million) format, while the associated clinical information was acquired through the UCSC Xena database [10] (https://xena.ucsc.edu/), the comprehensive details are presented in Table 1.

**Table 1:** Baseline table with THCA patients characteristics

Characteristics	Overall
Age, median (IQR)	46 (35, 57.75)
Gender, n (%)	
Female	187 (68.2%)
Male	87 (31.8%)
NStage, n (%)	
N1	151 (55.1%)
N0	94 (34.3%)
NX	29 (10.6%).
TStage, n (%)	
T1	51 (18.6%)
T2	94 (34.3%)
T3&4	129 (47.1%)
Stage, n (%)	
I	140 (51.1%)
IV	42 (15.3%)
II&III	92 (33.6%)

*THCA* thyroid carcinoma

Through the R package GEOquery [11] (Version 2.70.0) from GEO database [12] (https://www.ncbi.nlm.nih.gov/geo/) to download RAI-refractory papillary thyroid cancer dataset GSE151181 [13]. The samples of dataset GSE151181 were from Homo sapiens, with the tissue origin being the thyroid; the specific details are provided in Table 2. GPL23159 was selected as the chip platform for the GSE151181 dataset, which contains 17 samples of Radioactive Iodine After Papillary Thyroid Cancer (RAI After PTC) samples and 22 samples of Radioactive Iodine Before Papillary Thyroid Cancer (RAI Before PTC) samples. Only the RAI After PTC samples and the RAI Before PTC samples were included in this study.

**Table 2:** GEO microarray chip information

	GSE151181
Platform	GPL23159
Species	Homo sapiens
Tissue	Thyroid
Samples in PTC After RAI group	17
Samples in PTC Before RAI group	22
Reference	PMID: 33198784

*GEO* Gene Expression Omnibus, *PTC* papillary thyroid cancer, *RAI* radioactive iodine

At the same time, the single cell dataset GSE184362 [14] of RAI-Refractory PTC was downloaded from GEO database for single-cell analysis. The samples from the GSE184362 dataset originated from Homo sapiens, and the tissue source was the thyroid gland. The GPL24676 chip platform was employed for the GSE184362 dataset, consisting of 2 samples of RAI After PTC and 7 samples of RAI before PTC. Only all samples of the RAI After PTC and the RAI before PTC were included in this research.

The ubiquitin-related genes (URGs) were obtained from the GeneCards database [15] (https://www.genecards.org/). The GeneCards database offers comprehensive information on human genes. By utilizing “Ubiquitin” as the search term and applying filters to retain only those URGs categorized as “Protein Coding” with a “Relevance Score” exceeding 0.8, a total of 9831 URGs were identified. Furthermore, the set of URGs was searched in the published literature [16] on the PubMed website using the keyword “Ubiquitin” (https://pubmed.ncbi.nlm.nih.gov/), resulting in a total of 11 URGs. Following the integration and deduplication of the data, a total of 9832 URGs were obtained. For further details, please refer to Table S1 and “GeneCards-SearchResults.csv”.

Finally, the R package limma [17] (Version 3.58.1) was used to standardize the GSE151181 dataset. This process included the utilization of annotation probes, normalization, and other techniques. Subsequently, the expression values of the dataset were compared before and after standardization through the use of a box plot.

### RAI treatment-related URDEGs in thyroid cancer

Based on the sample categorization within the TCGA-THCA dataset, we segregated the samples into two distinct groups: those exhibiting RAI Sensitivity in PTC and those demonstrating RAI Resistance in PTC. Following this, differential expression analysis was performed on the genes within these two groups utilizing the DESeq2 [18] (Version 1.42.0). The criteria established for identifying differentially expressed genes necessitated log FC greater than 1.5 and adj. p of less than 0.05, corrected by the Benjamini–Hochberg (BH) method. In particular, genes exhibiting log FC > 1.5 and adj. *p* < 0.05 were identified as Up-regulated differentially expressed genes, whereas genes with log FC < − 1.5 and adj. *p* < 0.05 were Down-regulated differentially expressed genes. The analysis of differences was visualized using the ggplot2 package (Version 3.4.4) in R, specifically to create volcano plots.

To obtain URDEGs associated with RAI-refractory PTC of thyroid cancer, All DEGs with |log FC| > 1.5 and adj. *p* < 0.05 obtained by differential analysis in the dataset TCGA-THCA were interposed with URGs and drawn Venn diagram to obtain URDEGs. The results from the differential analysis were subsequently illustrated as a volcano plot, utilizing the pheatmap package (Version 1.0.12) in R.

### GO and KEGG enrichment analysis

A GO analysis [19] represents a widely utilized approach for carrying out large-scale functional annotation studies, encompassing aspects related to Biological Processes (BP), Cellular Components (CC), and Molecular Functions (MF). KEGG [20], on the other hand, serves as an extensive database that stores a wide range of information pertaining to genomes, biological pathways, diseases, and drugs. To achieve a more comprehensive understanding of the functional characteristics of URDEGs, we performed GO and KEGG analysis of URDEGs using the R package cluster Profiler [21] (Version 4.10.0). The criteria for item selection included a p-value of less than 0.05 and a FDR value (q-value) of less than 0.25. The p-value adjustment was performed using the Benjamini-Hochberg (BH) method.

### Establishment of prognostic risk model for thyroid cancer treated with RAI

To develop the prognostic risk model within the dataset TCGA-THCA, we used the R package survival (Version 3.5-7) to conduct a univariate Cox regression analysis utilizing clinical data to assess the impact of URDEGs on prognosis. To ascertain whether it serves as an independent prognostic factor, we first performed a univariate Cox regression analysis. Variables with a p-value less than 0.05 were then selected for further multivariate Cox regression analysis.

Subsequently, LASSO regression analysis was carried out using the R package glmnet [22] (Version 4.1-8) with family = “cox” as the parameter based on the URDEGs included in the univariate Cox regression analysis, and the number of cycles was set to 10. The Model Genes comprising prognostic risk model were identified. LASSO regression builds upon linear regression by incorporating a penalty (lambda × absolute value of slope), mitigating overfitting and enhancing model generalization. The results were illustrated via prognostic risk model and variable path plots. The RiskScore was computed using risk coefficients from LASSO regression, applying the given formula:

$${\mathrm{risk}}\;Score=\mathop \sum \limits_{i} Coefficient \; \left( {gen{e_i}} \right) * mRNA \; Expression \; \left( {gen{e_i}} \right)$$

### Prognostic analysis of prognostic risk model of RAI therapy for thyroid cancer

Time-dependent Receiver Operating Characteristic (ROC) Curve [23] is as a pivotal coordinate graphical analysis tool in the optimization of models, the discarding of inferior models, and the establishment of optimal cut-off points within a single model. The survival ROC R package (version 1.0.3) was used to produce ROC curves, which were generated based on the RiskScore and the Progression-Free Interval (PFI) derived from the prognostic risk model. Additionally, the Area Under the Curve (AUC) of these ROC curves was computed. The ROC curve was utilized to plot AUC, which served as a predictor for the 1-, 2-, and 3-year survival outcomes of thyroid cancer samples from the dataset TCGA-THCA that were RAI-Refractory PTC. Typically, the AUC ranges from 0.5 to 1, with values closer to 1 indicating superior diagnostic performance. An AUC greater than 0.5 suggests a trend towards promoting the event’s occurrence. An AUC between 0.5 and 0.7 signifies low accuracy, whereas between 0.7 and 0.9 it indicates moderate accuracy, and an AUC exceeding 0.9 denotes high accuracy. To analyze the difference in PFI between the High Risk and Low Risk groups in the dataset TCGA-THCA, The R package survival (Version 3.5-7) was used for Kaplan-Meier (KM) curve [24] analysis and KM curve was plotted on the basis of RiskScore.

The Forest Plot was employed to visually represent the results of both univariate and multivariate Cox regression analyses, highlighting the expression of the RiskScore alongside the clinical information incorporated into these analyses. A Nomogram [25] is a graphical tool that employs a series of non-overlapping line segments within a rectangular coordinate system to depict the functional relationship among multiple independent variables. Utilizing the R package rms (Version 6.7-1), we constructed a Nomogram based on the findings of multivariate Cox regression analysis, illustrating the correlation between the RiskScore and clinical data.

The Calibration Curve serves as a tool to assess the predictive performance of the model against actual outcomes by plotting the fit between observed probabilities and those predicted by the model across various scenarios. Through Calibration analysis, we generated the Calibration Curve to evaluate the precision and discriminatory power of the prognostic risk model that incorporates the RiskScore.

### Differential expression verification and ROC curve analysis

According to the RAI-Refractory PTC RiskScore of prognostic Risk model to express the median value put data sets the dataset TCGA-THCA samples were categorized into High Risk group and the Low-Risk group. To further investigate the expression variations of Model Genes between the High Risk and Low-Risk groups in the dataset TCGA-THCA, the group comparison diagram was generated based on the expression levels of Model Genes. Finally, the R package pROC [26] (Version 1.18.5) was utilized to generate the ROC curve and calculate the AUC for the Model Genes. This analysis was conducted to evaluate the diagnostic efficacy of the expression levels of these genes in predicting the occurrence of RAI-Refractory PTC.

Next, the RiskScore of RAI After PTC samples from the GSE151181dataset was analyzed using the risk coefficient. Based on the median expression of RiskScore, the RAI After PTC samples of the GSE151181 dataset were categorized into High Risk group and Low Risk group. To further explore the expression differences of Model Genes in two groups in the RAI After PTC samples of the GSE151181 dataset. The group comparison chart was created based on the expression levels of the Model Genes. In summary, the pROC R package (Version 1.18.5) was employed to visualize the ROC curve for these Model Genes and to calculate the AUC. This analysis aimed to evaluate the diagnostic performance of the expression levels in predicting RAI-refractory PTC.

### Quality control of single cell dataset

We utilized the “Create Seurat” [27] from the Seurat R package (Version 5.0.1) to import the Counts matrix for all samples in the GSE184362 dataset and to construct a Seurat object. The parameters were configured to include genes that were expressed in at least three cells and required a minimum of 200 genes expressed per cell. Because low-quality cells or empty droplets usually have very few genes, we filtered out cells with RNA number < 500, RNA number feature number < 250, log10GenesPerUMI < 0.8, and mitochondrial gene content > 20%.

Then, we pass the Normalize Data function of the GSE184362 dataset sequencing depth for standardization, standardization of methods for the default “Log Normalize”, The “vst” method was used to detect the top 2000 hypervariable genes of the dataset by calling the “Find Variable Features” function. We subsequently scaled the data using the “Scale Data” function to mitigate the influence of sequencing depth. Principal Component Analysis (PCA) was conducted to identify significant principal components, and the “Elbow Plot” function was employed to visualize the distribution of p-values. Ultimately, 30 significant principal components were selected for dimensionality reduction using Uniform Manifold Approximation and Projection (UMAP) analysis. To construct the k-nearest neighbors based on Euclidean distances within the initial PCA space, we utilized the default settings of the “Find Neighbors” function, incorporating the thirty notable principal component dimensions as parameters. By utilizing the “Find Clusters” function, we employed the “clustree” function to determine a resolution of 0.6, enabling the division of cells into distinct clusters. Finally, the “Run UMAP” function was applied for dimensionality reduction, facilitating visualization and exploration of the dataset.

### Cell type annotation and single-cell taxa differential genes

Initially, we annotated cell types in the GSE184362 dataset using the “SingleR [28]” function from the R package SingleR (Version 2.4.1), referencing the ImmGenData dataset. Subsequently, we employed the “Dot Plot” and “Feature Plot” functions to visualize the expression levels of Model Genes across different cell types. To identify genes with differential expression across various cell clusters, we utilized the “FindAllMarkers” function. This function compares the gene expression profile of each individual cell to the combined expression of all other cells, employing the Wilcoxon rank-sum test. We selected the top 10 differentially expressed genes from each cell cluster as the single-cell group Differentially Expressed Genes (scDEGs) for further analysis.

### AUCell analysis

AUCell could identify the single cell RNA sequence data in the cells with an active gene set. The R package AUCell [29] (Version 1.24.0) calculates AUC to assess whether a specific subset of the input gene set is enriched among the expressed genes in each individual cell. Analyzing the distribution of AUC scores across all cells facilitates the investigation of relative feature expression. Given that the scoring method is rank-based, AUCell is robust against variations in gene expression units and standardization processes. Additionally, since each cell is evaluated independently, the method can be readily applied to larger datasets and organized expression matrices. We focused on the top 10 differential genes for AUCell scoring and identified cell populations that exhibited high scores.

### Subgroup analysis of different cells

To further investigate the subgroups of SMC, we extracted these cells from the GSE184362 dataset. We then utilized the Seurat R package (Version 5.0.1) to perform normalization and clustering for dimensionality reduction once again. To enhance the distinction among the subgroup analyses, we utilized the “Dot Plot” and “Feature Plot” functions to visually represent the expression levels of Model Genes in SMC across different subgroups and their classifications.

To identify the DGEs among cell subsets, we used the “FindAllMarkers” function to compare the difference of gene expression between cells and other cell subsets by Wilcoxon rank sum test. In conclusion, we conducted cell type annotation for different cell subsets based on the differential genes identified between them.

### Gene Set Variation Analysis of cell subsets (GSVA)

GSVA [30] is a nonparametric, unsupervised method for evaluating gene set enrichment in microarray nuclear transcriptomes. It converts gene expression matrices from different samples into a format that highlights variations in gene expression between samples, allowing for a more robust assessment of specific gene set enrichment. To evaluate whether different pathways are enriched in different samples. To assess the enrichment of distinct pathways across various samples, we acquired the h.all.v7.4.symbols.gmt gene set from the Molecular Signatures Database [31] (MSigDB) (https://www.gsea-msigdb.org/gsea/msigdb). The expression values for all genes were grouped and averaged based on subsets of SMC. Finally, GSVA was conducted, and the resulting gene set variations were visualized using the R package pheatmap (Version 1.0.12).

### Statistical analysis

The data processing and analysis presented in this article were conducted using R software (version 4.3.0). In the absence of specific instructions, comparisons between two groups of continuous variables were evaluated for statistical significance through an independent Student’s t-test for normally distributed variables. For variables that were not normally distributed, the Mann-Whitney U test was utilized. For comparisons involving three or more groups, the Kruskal-Walls test method was employed. Spearman’s rank correlation coefficient was calculated to assess the correlation between different molecules. Unless otherwise stated, all statistical p-values were two-tailed, and statistical significance was defined as a p-value less than 0.05.

## Results

### Technology roadmap

**Fig. 1:**
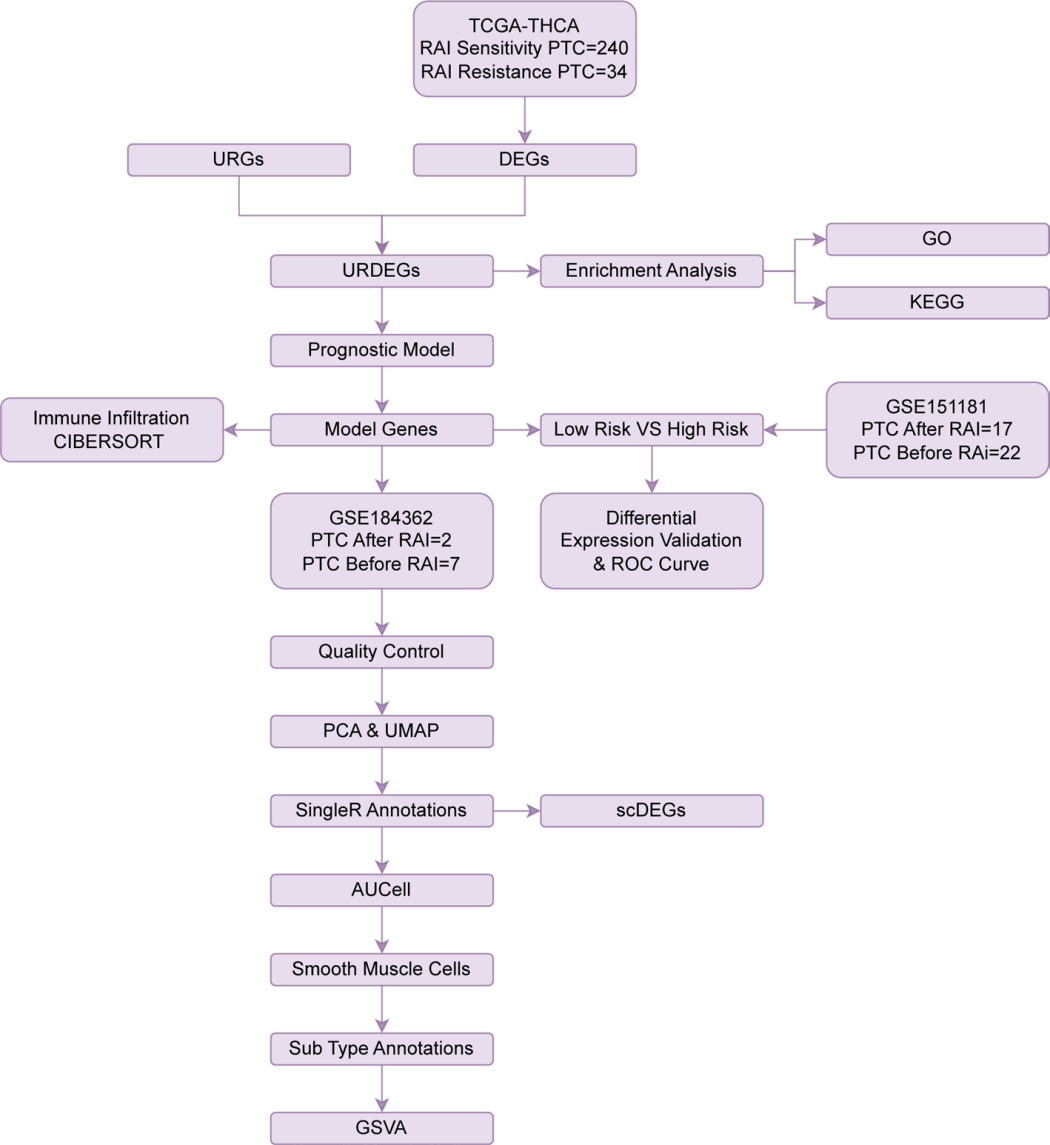
Flow chart for the comprehensive analysis of URDEGs

The technical roadmap for achieving the research objectives is illustrated in the following (Fig. 1).

### Standardization of RAI treatment dataset for thyroid cancer

**Fig. 2:**
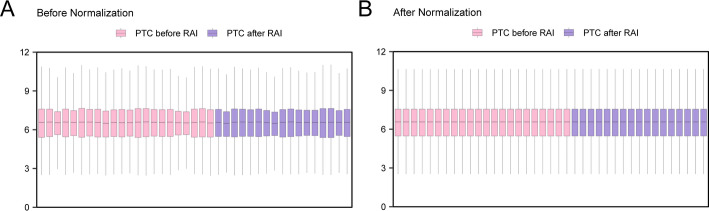
Normalization of GSE151181. **A** Boxplot of GSE151181 distribution in the dataset before normalization. **B** Boxplot of GSE151181 distribution of the data set after standardized processing. Purple is the sample RAI After PTC, pink is the sample RAI before PTC

First, through the R package limma GSE151181 for data set in standardization, standardization of annotation probes, normalized processing. Subsequently, the distribution boxplot (Fig. 2A, B) was utilized to compare the expression values of the GSE151181 dataset before and after normalization.

### Thyroid cancer associated RAI therapy differentially expressed genes related to ubiquitin

**Fig. 3:**
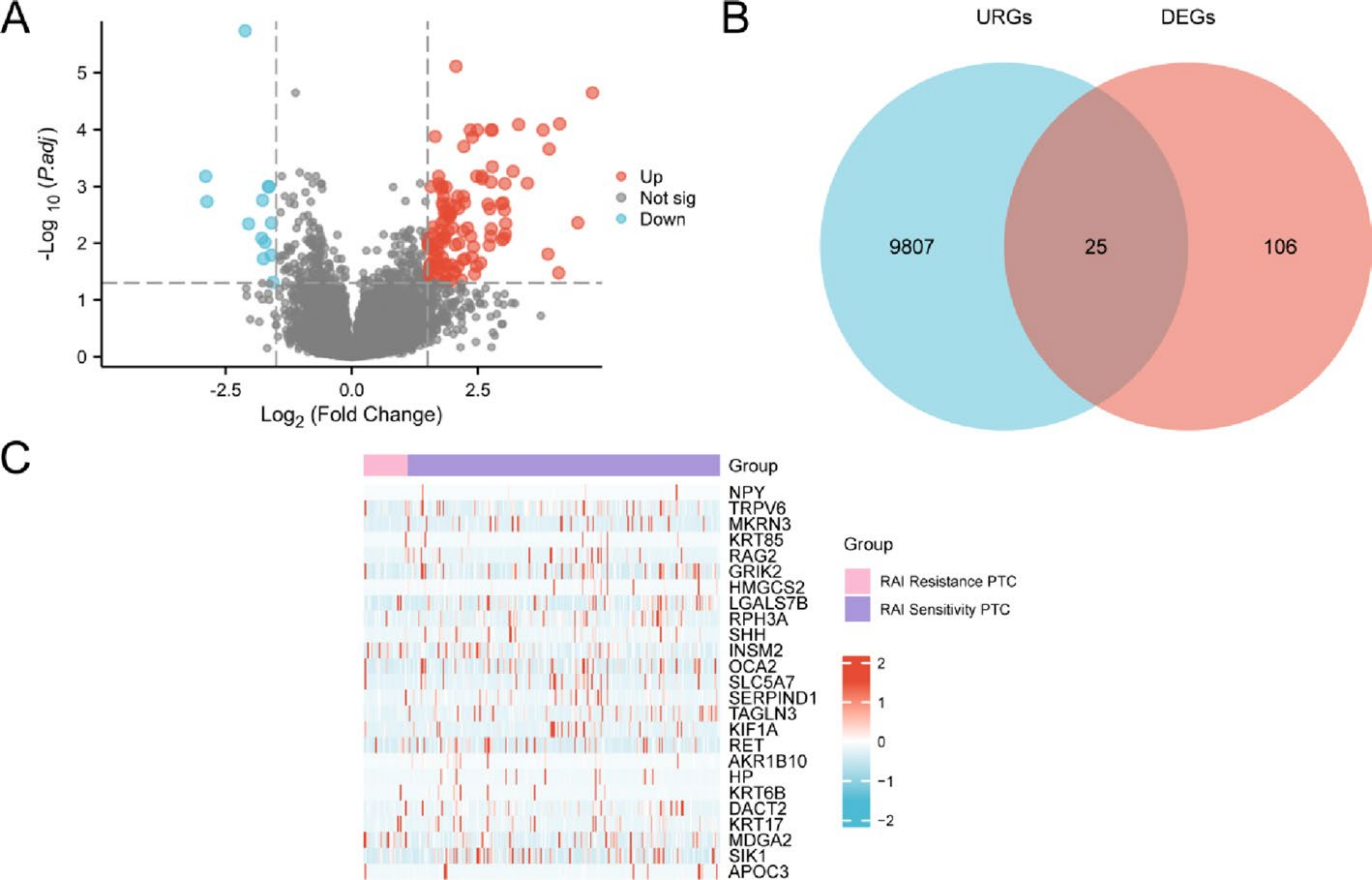
Differential gene expression analysis. **A** Volcano plot of differentially expressed genes analysis between RAI Sensitivity and RAI Resistance PTC groups in the dataset TCGA-THCA. **B** Venn diagram of DEGs and URGs in the dataset TCGA-THCA. **C** Heat map of URDEGs in the dataset TCGA-THCA. Pink is RAI Resistance PTC group and purple is RAI Sensitivity PTC group in heat map grouping. In the heat map, red represents high expression and blue represents low expression

The TCGA-THCA dataset was categorized into two groups: RAI Sensitivity PTC and RAI Resistance PTC. This classification enabled us to analyze the differences in gene expression values between the two groups within the TCGA-THCA datasetThe R package DESeq2 was employed to conduct differential analysis on the TCGA-THCA dataset, identifying differentially expressed genes (DEGs) between the two groups. The analysis revealed a total of 131 DEGs that met the criteria of |logFC| > 1.5 and adjusted *p* < 0.05. Among these, 118 genes were up-regulated (logFC > 1.5 and adj.*p* < 0.05), while 13 genes were down-regulated (logFC < − 1.5 and adj.*p* < 0.05). Based on the differential analysis results from this dataset, a volcano plot (Fig. 3A) was generated.

To identify URDEGs, we intersected all DEGs meeting the criteria of |logFC| > 1.5 and adj.*p* < 0.05 with URGs, resulting in a Venn diagram (Fig. 3B). This analysis yielded a total of 25 URDEGs, with detailed information provided in Table S2. Following the intersection results, we examined the expression differences of URDEGs within the TCGA-THCA dataset. The R package pheatmap was utilized to create a heatmap that visualizes the analysis outcomes (Fig. 3C).

### GO and KEGG enrichment analysis

**Fig. 4:**
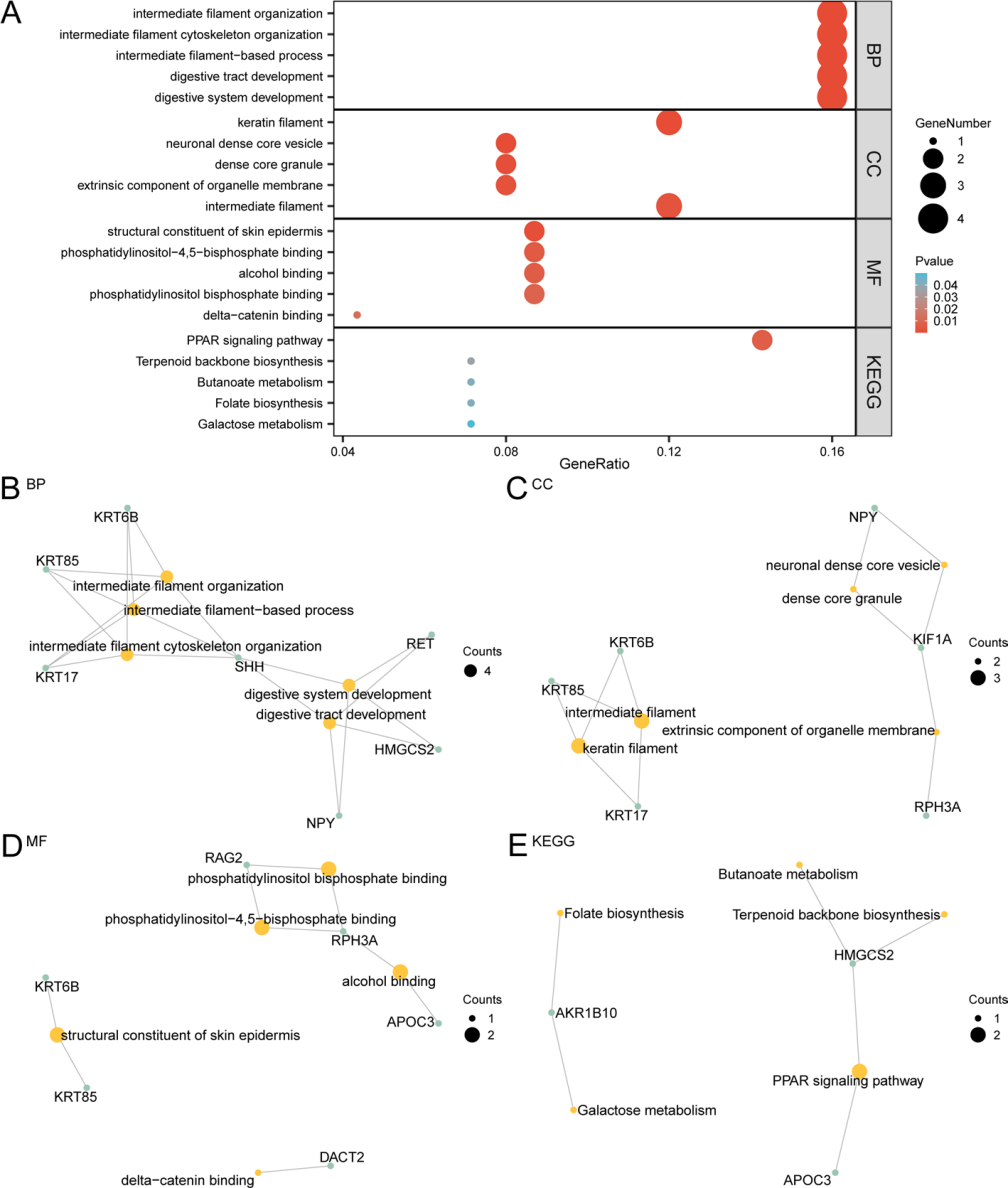
GO and KEGG enrichment analysis for URDEGs. **A** URDEGs GO and KEGG enrichment of bubble chart analysis results show: BP, CC, the MF and KEGG. The y coordinate for the GO terms and KEGG terms. **B**–**E** URDEGs GO and KEGG enrichment of network diagram analysis results show: BP (**B**), CC (**C**), MF (**D**) and KEGG (**E**). Orange nodes represent entries, green nodes represent molecules, attachment on behalf of the entry and molecular relationship. In the bubble plot, the size of each bubble denotes the count of genes, whereas the bubble color signifies the magnitude of the p-value. Specifically, a redder color indicates a smaller p-value, conversely, a bluer hue signifies a larger p-value. For the purposes of GO and KEGG enrichment analysis, the screening criteria were established as follows: a p-value threshold of less than 0.05 and an FDR value (or q-value) threshold of less than 0.25

GO and KEGG enrichment analysis were used to further explore the BP, CC, and CC of 25 URDEGs. The relationship between MF and KEGG and RAI-Refractory PTC of thyroid cancer was explored. The 25 URDEGs were analyzed for GO and KEGG enrichment, and the detailed results are presented in Table 3. The analysis revealed that the 25 URDEGs were primarily enriched in processes associated with intermediate filament organization in RAI-Refractory PTC. Intermediate filament cytoskeleton organization, intermediate filament-based process, digestive tract development, digestive system development and other BP; keratin filament, neuronal dense core vesicle, dense core granule, extrinsic component of organelle membrane, intermediate filament and other CC; structural constituent of skin epidermis, phosphatidylinositol-4,5-bisphosphate binding, alcohol binding, phosphatidylinositol bisphosphate binding, delta-catenin binding and other MF. It was also enriched in PPAR signaling pathway, Terpenoid backbone biosynthesis, Butanoate metabolism, Folate biosynthesis, Galactose metabolism and other biological pathways (KEGG). The results of the GO and KEGG enrichment analyses were illustrated using bubble plots (Fig. 4A). Additionally, network diagrams were created for biological processes (BP), cellular components (CC), molecular functions (MF), and KEGG pathways (Fig. 4B–E). These diagrams indicate the corresponding annotations of molecules, with larger nodes representing a greater number of associated molecules.

**Table 3:** Result of GO and KEGG enrichment analysis for URDEGs

Ontology	ID	Description	Gene ratio	Bg ratio	*p* value
BP	GO:0045109	Intermediate filament organization	4/25	73/18,614	2.59 e−06
BP	GO:0045104	Intermediate filament cytoskeleton organization	4/25	92/18,614	6.53 e−06
BP	GO:0045103	Intermediate filament-based process	4/25	93/18,614	6.82 e−06
BP	GO:0048565	Digestive tract development	4/25	134/18,614	2.89 e−05
BP	GO:0055123	Digestive system development	4/25	146/18,614	4.04 e−05
CC	GO:0045095	Keratin filament	3/25	97/19,518	2.53 e−04
CC	GO:0098992	Neuronal dense core vesicle	2/25	28/19,518	5.83 e−04
CC	GO:0031045	Dense core granule	2/25	41/19,518	1.25 e−03
CC	GO:0031312	Extrinsic component of organelle membrane	2/25	51/19,518	1.93 e−03
CC	GO:0005882	Intermediate filament	3/25	214/19,518	2.50 e−03
MF	GO:0030280	Structural constituent of skin epidermis	2/23	36/18,369	9.21 e−04
MF	GO:0005546	Phosphatidylinositol-4,5-bisphosphate binding	2/23	82/18,369	4.69 e−03
MF	GO:0043178	Alcohol binding	2/23	88/18,369	5.38 e−03
MF	GO:1,902,936	Phosphatidylinositol bisphosphate binding	2/23	107/18,369	7.85 e−03
MF	GO:0070097	Delta-catenin binding	1/23	10/18,369	1.25 e−02
KEGG	hsa03320	PPAR signaling pathway	2/14	75/8776	6.14 e−03
KEGG	hsa00900	Terpenoid backbone biosynthesis	1/14	23/8776	3.61 e−02
KEGG	hsa00650	Butanoate metabolism	1/14	27/8776	4.23 e−02
KEGG	hsa00790	Folate biosynthesis	1/14	27/8776	4.23 e−02
KEGG	hsa00052	Galactose metabolism	1/14	32/8776	4.99 e−02

*GO* Gene Ontology, *BP* biological process, *CC* cellular component, *MF* molecular function, *KEGG* Kyoto Encyclopedia of Genes and Genomes, *URDEGs* ubiquitin-related differentially expressed genes

### Construction of prognostic risk model for thyroid cancer treated with RAI

**Fig. 5:**
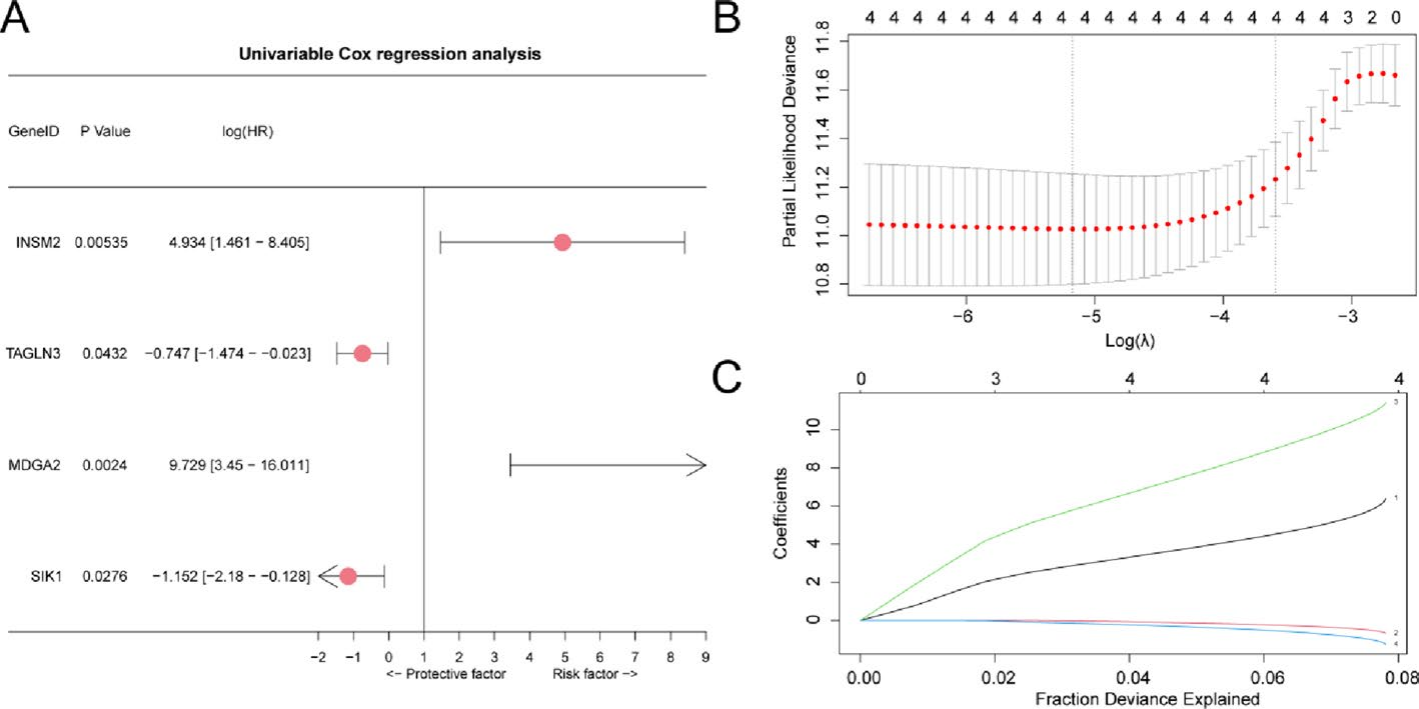
Cox regression analysis. **A** Forest Plot of 4 URDEGs in univariate Cox regression model. **B**–**C** Plots of prognostic risk model (**B**) and variable trajectories (**C**) from LASSO regression model. LASSO, further Absolute Shrinkage and Selection Operator

In order to build the RAI-Refractory PTC prognostic risk model, we applied 25 URDEGs related to single factor Cox regression analysis, And the single factor analysis of all the p value < 0.05 variables and through the Forest figure (Forest Plot) of visual display (Fig. 5A). Results show that the single factor in the Cox regression model, four URDEGs are statistically significant (p value < 0.05), respectively is: *INSM2*, *TAGLN3*, *MDGA2*, *SIK1*. In order to further determine the single factor gene in THCA Cox regression model in the RAI-Refractory PTC prognostic value, LASSO regression analysis and build LASSO regression model. By mapping the LASSO regression model (Fig. 5B) and LASSO variable trajectory diagram (Fig. 5C) of visual display. The analysis revealed that the LASSO regression model comprised four genes, specifically *INSM2*,* TAGLN3*,* MDGA2*,* SIK1*, which were selected through the LASSO regression procedure. RiskScore computation formula is as follows:

$$Risk\;Score=INSM2\;TAGLN3** \left( {5.95} \right)+\left( {0.552} \right)+\left( {11} \right)+SIK1\;MDGA2** \left( {1.07} \right)$$

### Prognostic validation of the model

#### Model discriminatory ability (time-dependent ROC analysis)

**Fig. 6:**
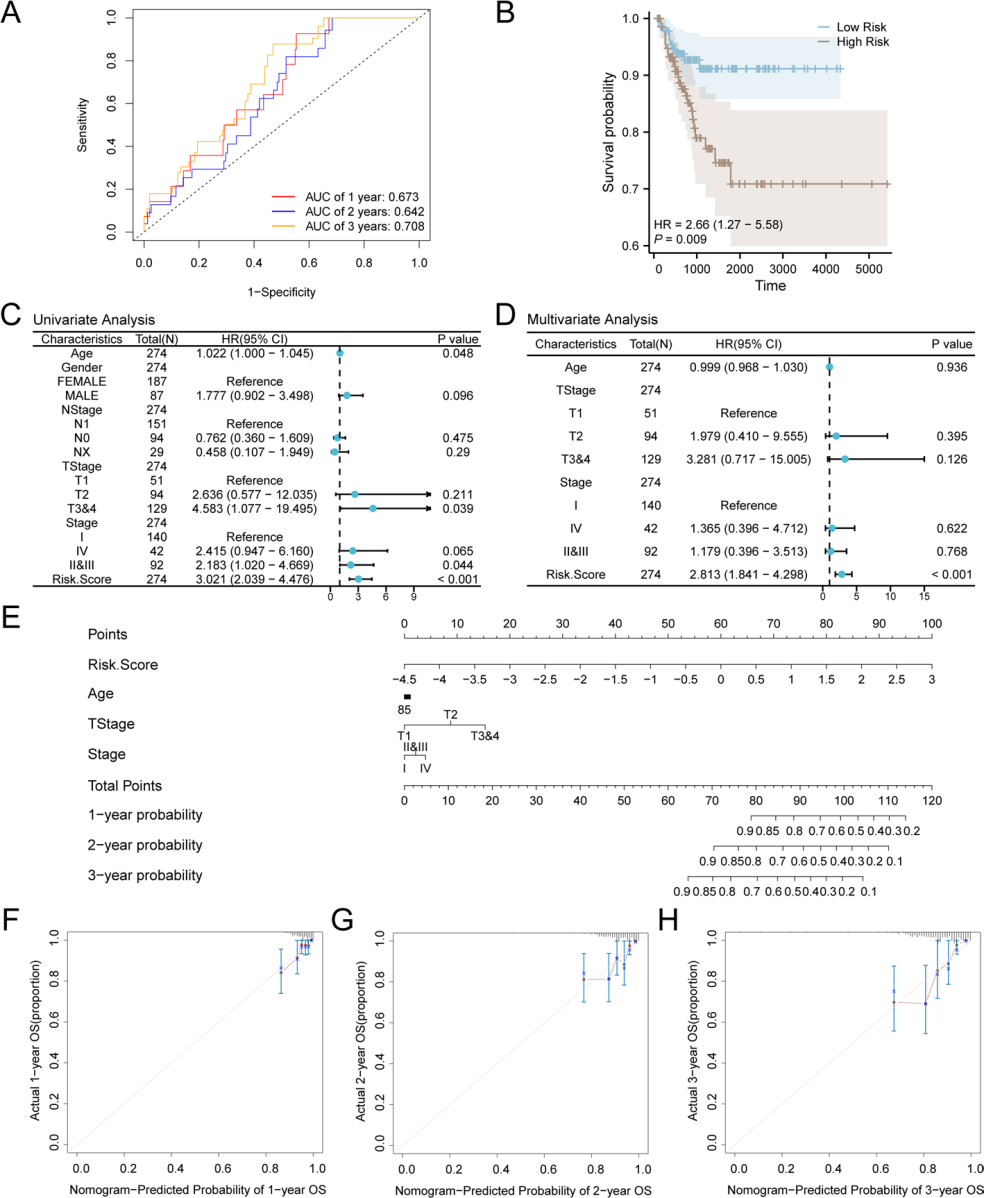
Prognostic analysis. **A** Time-dependent ROC curve of the dataset TCGA-THCA. **B** RiskScore of high and low group and the RAI - Refractory PTC samples of PFI between the prognosis of KM curve. **C**, **D** RiskScore and clinical information in single factor Cox regression model (**C**) and Multivariate Cox regression model (**D**) in the Forest figure (Forest Plot). **E** RiskScore and clinical information in a Multivariate Cox regression model (Nomogram) columns in the chart. **F**–**H** RAI–Refractory PTC prognostic risk model of 1 year (**F**), **G** 2 years, 3 years (**H**) Calibration Curve

First of all, we mapped the data sets the dataset TCGA-THCA time depend on the ROC curve (Fig. 6A). Our findings indicated that the prognostic risk model of the RAI-Refractory PTC had low accuracy at 1 and 2 years (0.7 > AUC > 0.5) and Fair Precision at 3 years (0.9 > AUC > 0.7).

#### Survival analysis and independent prognostic value

In addition, we also based RAI therapy for RAI-Refractory in the RiskScore Combined dataset TCGA-THCA PFI of PTC samples was analyzed by prognostic KM curve based on median grouping (Fig. 6B). The results showed that High Risk group and Low Risk group and the dataset TCGA-THCA RAI-Refractory PTC samples of PFI between highly statistically significant (p value < 0.01).

Then we based on RiskScore value in the group with the RAI-Refractory PTC samples of PFI and clinical information of single factor Cox regression analysis, Variables and screening of p value < 0.10 Multivariate Cox regression analysis. The univariate and multivariate Cox regression analysis findings were depicted via Forest Plots (Fis. 6 C, D), with detailed information presented in Table 4. The results from both the univariate and the multivariate Cox regression analyses revealed that the RiskScore exhibited statistical significance (*p* < 0.05).

**Table 4:** Results of Cox analysis

Characteristics	Total(*N*)	Univariate analysis		Multivariate analysis	
		HR (95% CI)	*P* value	HR(95% CI)	*P* value
Age	274	1.022 (1.000–1.045)	0.048	0.999 (0.968–1.030)	0.936
Gender	274				
Female	187	Reference			
Male	87	1.777 (0.902–3.498)	0.096		
NStage	274				
N1	151	Reference			
N0	94	0.762 (0.360 1.609)	0.475		
NX	29	0.458 (0.107–1.949)	0.290		
TStage	274				
T1	51	Reference		Reference	
T2	94	2.636 (0.577–12.035)	0.211	1.979 (0.410 9.555)	0.395
T3&4	129	4.583 (1.077 19.495)	0.039	3.281 (0.717–15.005)	0.126
Stage	274				
I	140	Reference		Reference	
IV	42	2.415 (0.947–6.160)	0.065	1.365 (0.396–4.712)	0.622
II&III	92	2.183 (1.020–4.669)	0.044	1.179 (0.396–3.513)	0.768
RiskScore	274	3.021 (2.039–4.476)	< 0.001	2.813 (1.841–4.298)	< 0.001

*HR* Hazard ratio, general HR > 1 indicates that the variable is a risk factor, and HR < 1 is a protective factor. Univariate p values < 0.1 were included in the analysis

#### Development and validation of a clinical nomogram

To further investigate the value of a prognostic risk model for RAI-refractory papillary thyroid carcinoma (PTC), we developed a nomogram based on the findings from both univariate and multivariate Cox regression analyses. This nomogram illustrates the relationship between the RiskScore and three clinical factors within samples of RAI-refractory PTC (see Fig. 6E). The findings indicate that the RiskScore has a substantially greater utility in the prognostic risk model for RAI-refractory PTC compared to other variables. Age for the RAI-Refractory PTC the prognosis of the effectiveness of risk model was obviously lower than other variables of utility.

In addition, we calibrated the prognostic risk model of the RAI-Refractory PTC at 1, 2, and 3 years and drew a Calibration curve (Fig. 6F–H). The Calibration Curve features the survival probability predicted by the model on the horizontal axis and the actual survival probability on the vertical axis. This curve illustrates that the model’s predictions at various time points align more closely with the ideal gray line, signifying improved predictive accuracy at those times. The results indicate that the prognostic risk model for RAI-refractory PTC demonstrated optimal clinical prediction accuracy for a 3-year survival period.

### Differential expression verification and ROC curve analysis between high and low risk groups

**Fig. 7:**
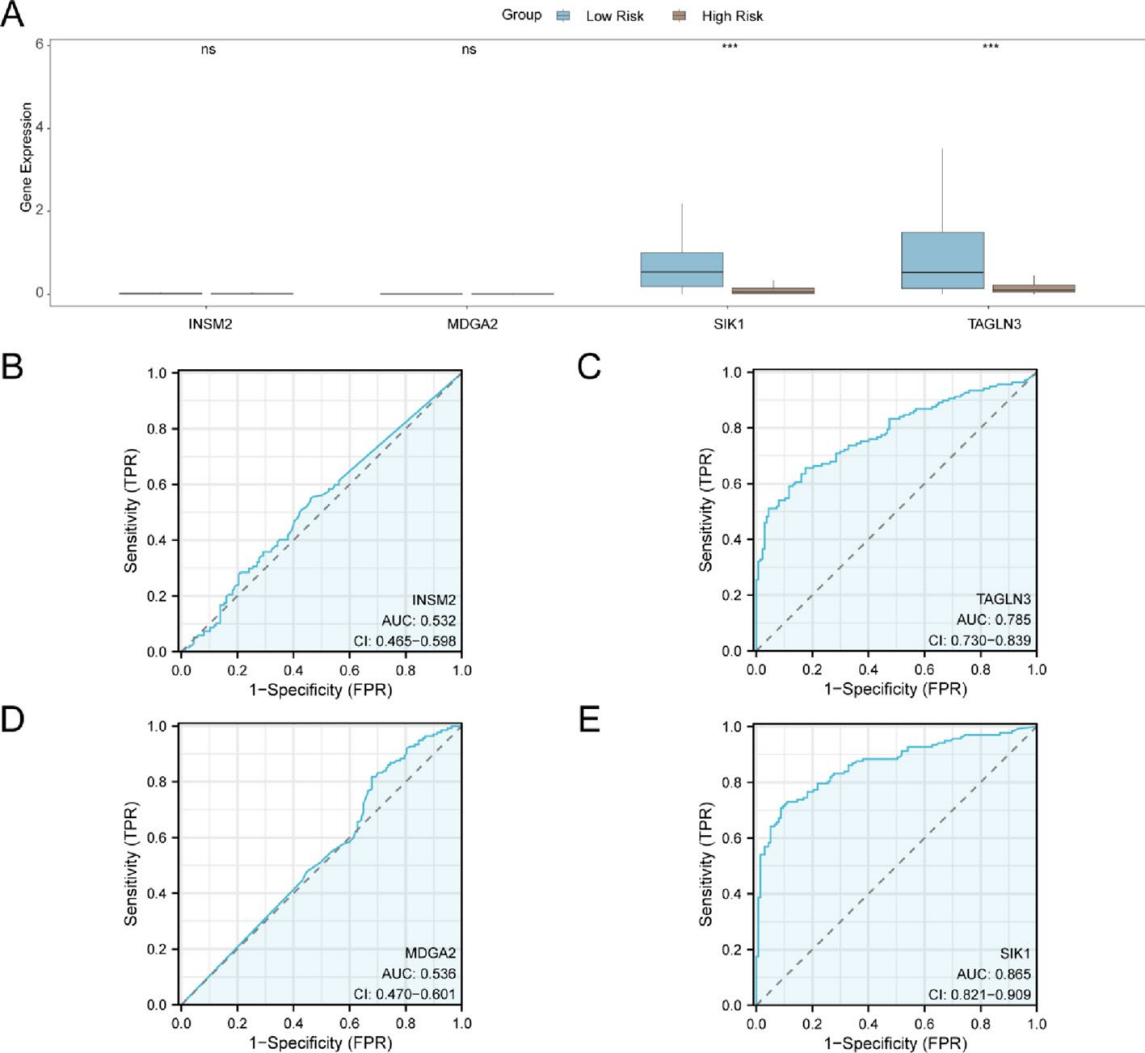
Differential expression validation and ROC curve analysis. **A** Model Genes in dataset TCGA-THCA in High Risk group and the Low Risk group in the comparison group. **B**–**E** Model Genes *INSM2* (**B**), *TAGLN3* (**C**), *MDGA2* (**D**) and *SIK1* (**E**) in dataset TCGA-THCA) in the ROC curve. ns represents p value ≥ 0.05, which is not statistically significant; *** represents p value < 0.001, highly statistically significant. Brown represents the High Risk group, and blue represents the Low Risk group. *TPR* true positive rate, *FPR* False positive rate

The samples of the dataset TCGA-THCA were categorized into High Risk group and Low Risk group according to the median expression value of RiskScore of the prognostic risk model of the RAI-Refractory PTC. To investigate the expression differences of Model Genes in the dataset TCGA-THCA (Fig. 7A) by grouping comparison chart shows four Model Genes in dataset TCGA-THCA in High Risk group and the Low Risk of the results of the analysis of difference in the expression amount of High and Low. The results of the differential analysis revealed that the expression levels of the two Model Genes in the High Risk group and the Low-Risk group in the dataset TCGA-THCA were highly statistically significant (p value < 0.001), respectively: *TAGLN3*, *SIK1*. Subsequently, the R package pROC was used to draw ROC curves (Fig. 7B–E) based on the expression of Model Genes (MG) in the dataset TCGA-THCA. The ROC curve showed that, the expression levels of two Model Genes *TAGLN3* and *SIK1* in the dataset TCGA-THCA showed certain accuracy in the classification of High Risk group and Low Risk group (0.7 < AUC < 0.9). However, the overall AUC value remains at a moderate level, indicating that the model’s predictive performance could be further improved. The expression levels of two other model genes, INSM2 and MDGA2, in the TCGA-THCA dataset showed lower accuracy in classifying the high-risk and low-risk groups (0.5 < AUC < 0.7).

**Fig. 8:**
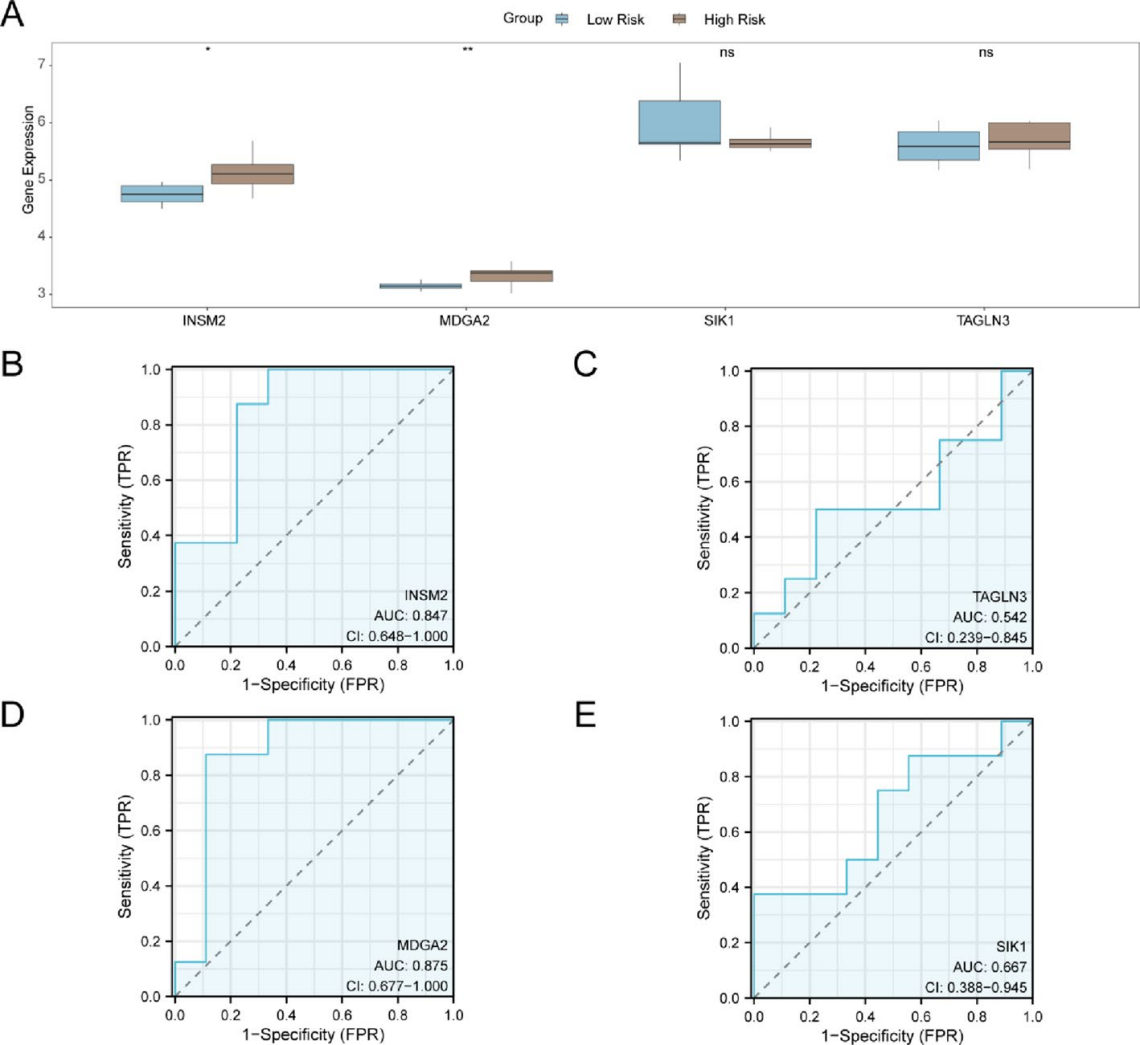
Differential expression validation and ROC curve analysis. **A** The group comparison diagram of Model Genes in the High Risk group and Low Risk group in the samples of RAI After PTC in dataset GSE151181. **B**–**E** ROC curves of Model Genes *INSM2* (**B**), *TAGLN3* (**C**), *MDGA2* (**D**) and *SIK1* (**E**) in RAI After PTC samples from dataset GSE151181. ns represents p value ≥ 0.05, which was not statistically significant; * represents p value < 0.05, indicating statistical significance; * * on behalf of the p value < 0.01, highly statistically significant. Brown for High Risk group, blue represents the Low Risk group

Subsequently, the RiskScore for RAI-refractory PTC samples in the GSE151181 dataset was calculated based on the risk coefficients derived from LASSO regression analysis. Using the median RiskScore value, the samples were categorized into High Risk and low-risk groups. To examine the expression variations of model genes in the RAI After PTC samples, the group comparison (Fig. 8A) shows the four Model Genes in the High Risk group and Low risk group of the RAI After PTC samples in the GSE151181 dataset. The differential analysis results indicated that the expression levels of two model genes (INSM2, MDGA2) were statistically significant (*p* < 0.05). Following this, we utilized the R package pROC to generate the ROC curve (Fig. 8B–E) based on the expression of these model genes in the RAI-refractory PTC samples from the GSE151181 dataset. The ROC curve demonstrated that the expression levels of the two model genes INSM2 and MDGA2 displayed a certain degree of accuracy in distinguishing between the High Risk and low-risk groups (0.7 < AUC < 0.9). The classification accuracy of the High Risk and Low Risk groups based on the expression levels of two model genes TAGLN3 and SIK1 was found to be relatively low, with an AUC value ranging from 0.5 to 0.7.

### High and low risk group of immune infiltration analysis (CIBERSORT)

**Fig. 9:**
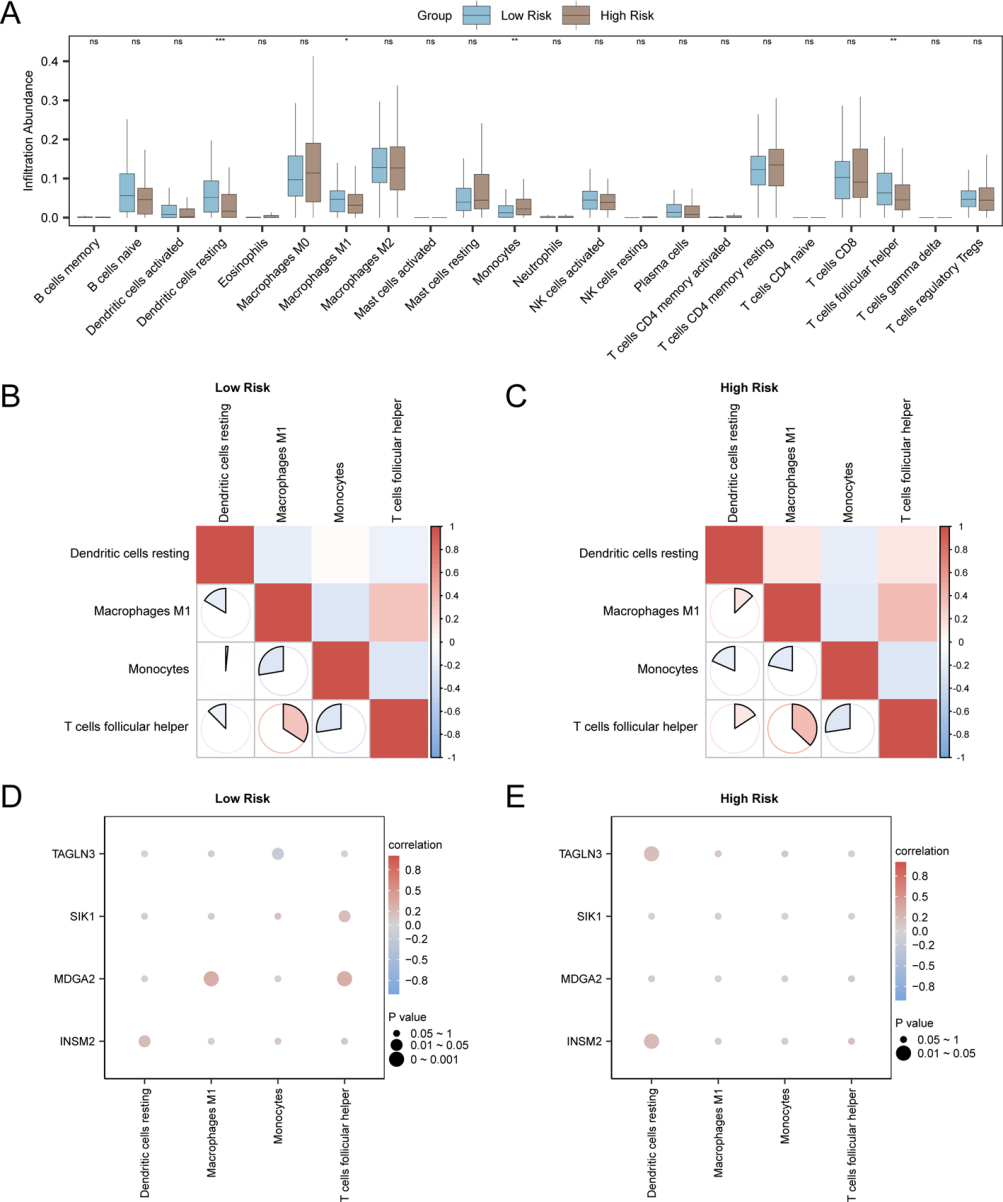
Risk group immune infiltration analysis by CIBERSORT algorithm. **A** The grouping comparison figure of immune cells in the Low Risk group and High Risk group of the dataset TCGA-THCA. **B**, **C** Correlation heat map of immune cells in the Low Risk group (**B**) and High Risk group (**C**) of the dataset TCGA-THCA. Bubble plot of correlation between immune cell infiltration abundance and Model Genes in the Low Risk group (**D**) and High Risk group (**E**) of dataset TCGA-THCA. * represents p value < 0.05, statistically significant; ** represents p value < 0.01, highly statistically significant; *** represents p value < 0.001 and highly statistically significant. The absolute value of the correlation coefficient (r value) below 0.3 was considered as weak or no correlation, and the r value between 0.3 and 0.5 was considered as weak correlation. Blue is the Low Risk group, and brown is the High Risk group. Red is the positive correlation, blue is the negative correlation. The depth of the color represents the strength of the correlation

Using the dataset TCGA-THCA, the immune infiltration abundance of 22 immune cells was calculated by CIBERSORT algorithm. First of all, according to the result of immune infiltration analysis, through the group comparison chart shows immune cells infiltrating abundance expression differences in different groups. The comparison chart presented in Fig. 9A reveals statistically significant differences (*p* < 0.05) among four types of immune cells, specifically: follicular helper T cells, monocytes, M1 macrophages, and resting dendritic cells.

Subsequently, the correlation heatmaps in Fig. 9B and C depict the correlation results for the infiltration abundance of four immune cell types within the TCGA-THCA dataset. The findings indicate that, within the Low Risk group of the TCGA-THCA dataset, the majority of immune cells exhibit correlations. Notably, follicular helper T cells and M1 macrophages displayed a positive correlation (*r* = 0.341, *p* < 0.05). In the High Risk group, a correlation was observed among most immune cells, with follicular helper T cells and M1 macrophages exhibiting a positive correlation (*r* = 0.371, *p* < 0.05). To conclude, the correlation between the model genes and the abundance of immune cell infiltration was visualized using a correlation bubble plot (Fig. 9D and E). According to the correlation bubble plot findings, the majority of immune cells within the Low Risk group of the TCGA-THCA dataset exhibited correlation. Among these, the gene MDGA2 displayed a positive correlation with M1 macrophages, with an r value of 0.306 and a p value below 0.05. A positive correlation was evident among the majority of immune cells within the High Risk group, with the resting Dendritic cells (DCs) exhibiting a positive correlation with the INSM2 gene, yielding an r value of 0.205 and a p value below 0.05.

However, it is important to note that CIBERSORT uses bulk RNA expression data, which differs significantly from single-cell RNA sequencing in terms of sample types, sequencing platforms, and data structures. Direct quantitative comparisons across platforms could introduce biases. Therefore, the CIBERSORT results reflect overall immune infiltration trends rather than precise cell proportions.

### Quality control of single-cell datasets

**Fig. 10:**
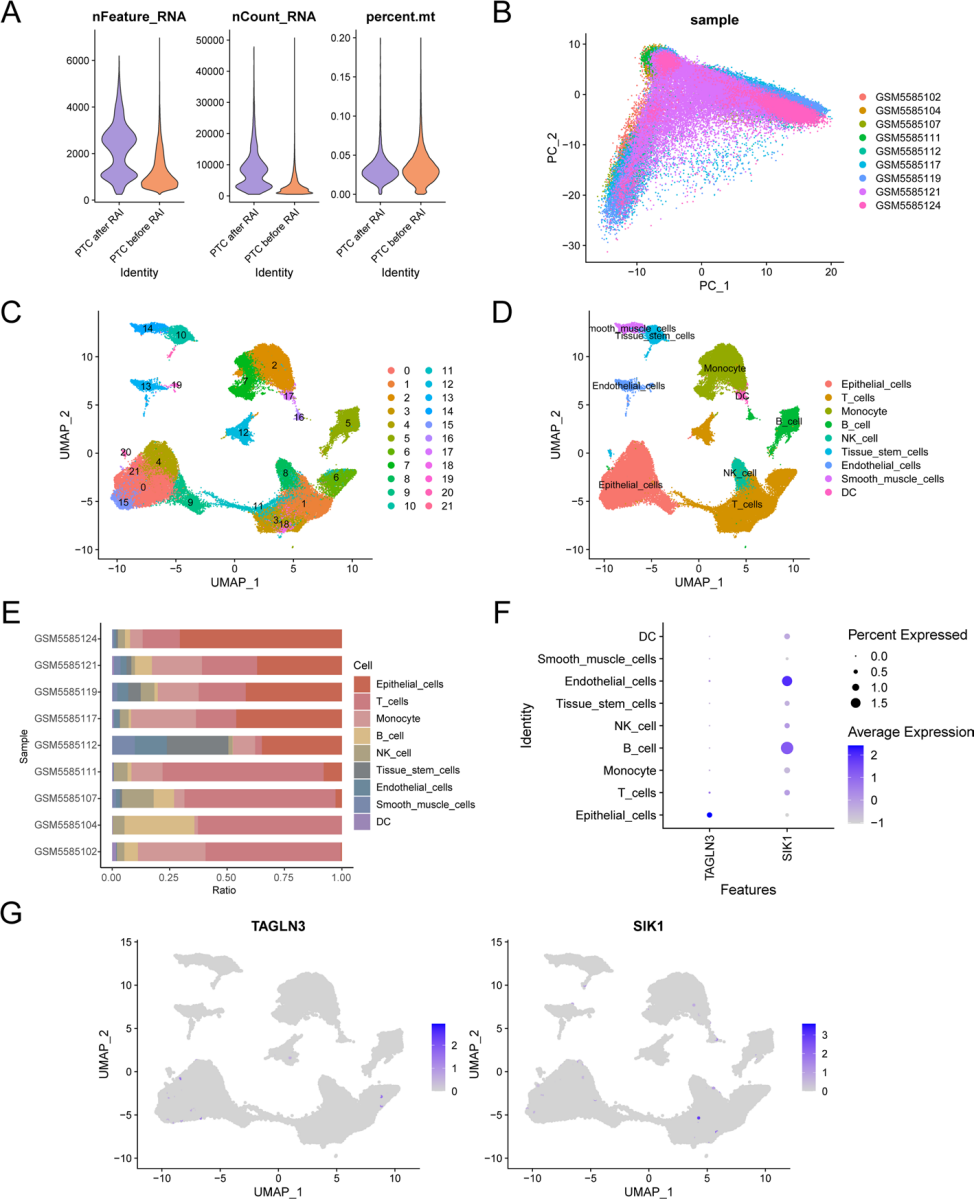
Quality Control of GSE184362. **A** Violin plot of gene expression for dataset GSE184362. **B** Visualization of PCA analysis of cell expression in different samples. **C** 74,497 cells were clustered into 22 cell clusters by UMAP. **D** Annotation of cells into nine cell types via the R package singleR: Epithelial Cells, T Cells mononuclear Cells, Monocyte, B Cells, NK cell, Tissue Stem Cells. Endothelial cells, SMC DC. **E** Bar graph of the proportion of cells in different samples. **F** Visualization of bubble plots of expression levels of two Model Genes. Darker colors indicate higher expression levels, and larger circles indicate higher percentages of genes expressed within the cell population. **G** UMAP diagram shows the expression levels of the two Model Genes in the single cell dataset

We utilized the “CreateSeuratObject” function of the R package Seurat to import 2 samples of the RAI After PTC and 7 samples of the RAI before PTC from the GSE184362 dataset Counts of sample matrix and create for Seurat object, parameter is set to at least three cells in each cell of the expression of genes and gene expression, at least 200 violin (Fig. 10A) show each cell number of genetic traits (nFeature RNA), Number of genes (nCount_RNA), average number of genes per UMI (log10GenesPerUMI). Next, we performed quality control on the GSE184362 dataset, filtering cells with RNA number < 500, RNA number characteristic number < 250, log10GenesPerUMI < 0.8, and mitochondrial gene content > 20% [32, 33] to obtain 74,497 cells (see beforeQC.png and afterQC.png). By PCA analysis visual cells expressed in different samples (Fig. 10B). Then, UMAP dimensionality reduction was applied for visualization. When the resolution was set to 0.6, 74,497 cells were successfully classified into 22 independent clusters (Fig. 10C). In our study, we utilized the R package SingleR to identify a total of nine distinct cell types within the clusters, as shown in Fig. 10D. These cell types include Epithelial Cells, T Cells, Mononuclear Cells, Monocytes, B Cells, Natural Killer Cells, Tissue Stem Cells, Endothelial Cells (EC), Smooth Muscle Cells (SMC), and DC. The proportion of cells between different samples (Fig. 10E) is visualized by the bar chart. The results indicated significant differences in the cell proportions among the various samples. Then, through the bubble chart (Fig. 10F) and UMAP (Fig. 10G) show two models respectively Model Genes in single-celled expression level of the data set. The results demonstrated that the Model Genes *TAGLN3 and SIK1* were predominantly enriched in Epithelial Cells and T Cells.

### Differential genes and aucell analysis of single-cell taxa

**Fig. 11:**
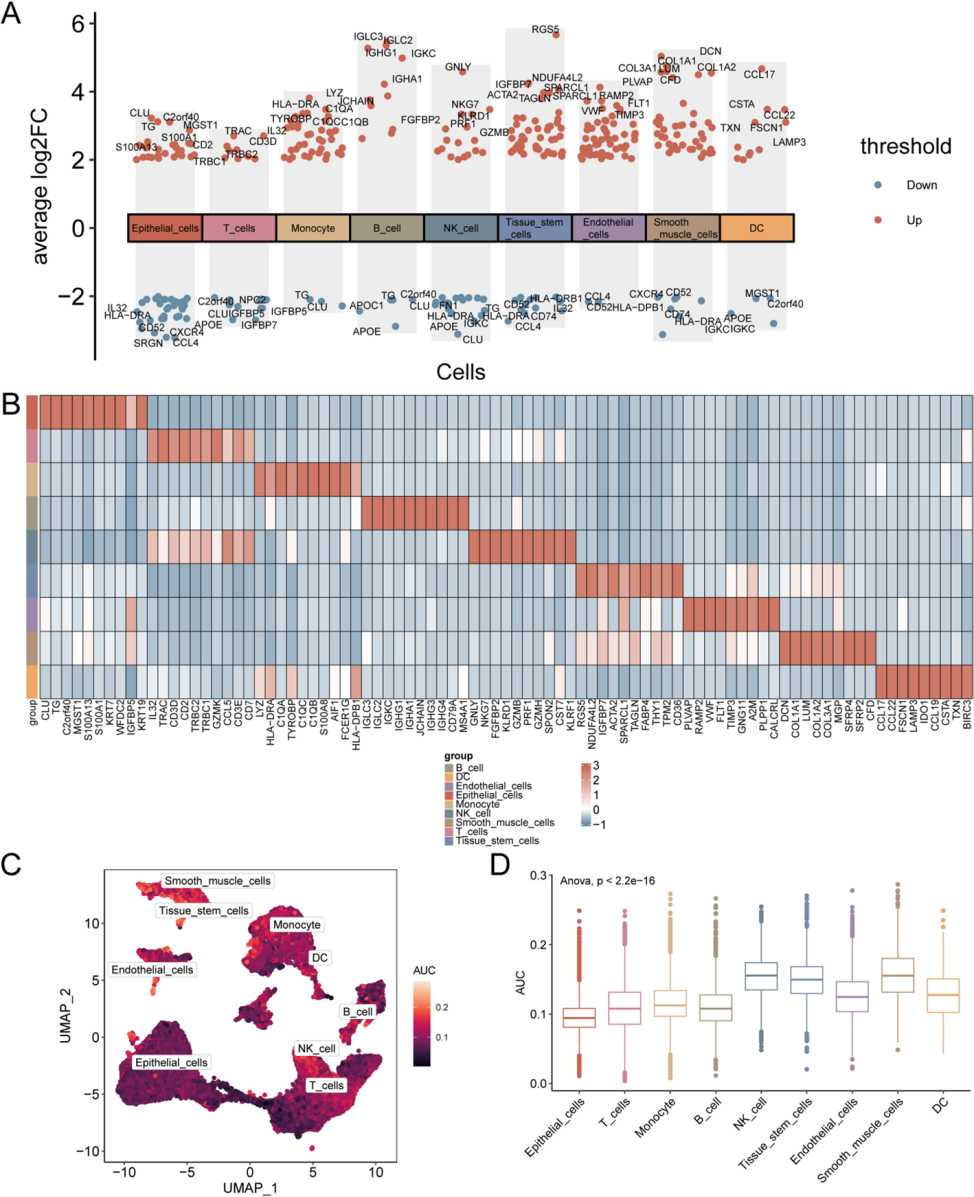
Differential gene expression and AUCell analysis for GSE184362. **A** Cell difference genes volcanic figure. Red for the cell gene expression level, blue for the cell gene expression downgrade. **B** Heat map of expression of scDEGs between cells. **C**, **D** The AUC score of scDEGs was visualized in UMAP map (**C**) and group comparison map (**D**) between different cell clusters. A lighter color of the UMAP map indicates a higher AUC score

We calculated the differential genes between cells through the R package FindAllMarkers, and took |log FC| > 2.00 and adj. *p* < 0.05 as the threshold to obtain the differential genes between cells and draw the volcano diagram (Fig. 11A). Then, by using heat maps show the top 10 up-regulated genes expression in the cell (Fig. 11B). Results show that the *CLU*,* TG*,* C2orf40*,* MGST1*,* S100A13*,* S100A1*,* KRT7*,* WFDC2*,* IGFBP5*,* KRT19* genes mainly in Epithelial Cells in the expression; *IL32*,* TRAC*,* CD3D*,* CD2*,* TRBC2*,* TRBC1*,* GZMK*,* CCL5*,* CD3E and CD7* were mainly expressed in T cells. *LYZ*,* HLA-DRA*,* C1QA*,* TYROBP*,* C1QC*,* C1QB*,* S100A8*,* AIF1*,* FCER1G*,* HLA-DPB1* were mainly expressed in monocytes. *IGLC3*,* IGLC2*,* IGKC*,* IGHG1 IGHA1*,* JCHAIN*,* IGHG3*,* IGHG4*,* CD79A*,* MS4A1* genes such as the main expression in B cell (B cell); *GNLY NKG7*,* FGFBP2 KLRD1*,* GZMB*,* PRF1*,* GZMH*,* SPON2*,* CST7*,* KLRF1* genes such as main expressed in NK cell; *RGS5*,* NDUFA4L2*,* IGFBP7*,* ACTA2*,* SPARCL1*,* TAGLN*,* FABP4*,* THY1*,* TPM2*,* CD36* genes mainly in organizations such as Stem Cells in the expression; *PLVAP*,* SPARCL1 RAMP2*,* VWF*,* FLT1 TIMP3*,* GNG11*,* A2M*,* PLPP1*,* CALCRL* genes mainly in Endothelial cells in the expression; *DCN*,* COL1A1*,* LUM*,* COL1A2*,* COL3A1*,* MGP*,* SFRP4*,* SFRP2*,* IGFBP7*,* CFD* genes mainly in SMC in the expression; *CCL17* and *CCL22*,* FSCN1 LAMP3 IDO1*,* CCL19*,* CSTA*,* TXN*,* HLA DPB1*, such as *BIRC3* genes mainly expressed in DC. AUCell we use R packet to every cell in the dataset GSE184362 might need to up-regulated Genes. The expression of scDEGs was scored and visualized by UMAP (Fig. 11C) and group comparison (Fig. 11D). The findings revealed that SMC exhibited the highest AUC score.

### Subgroup analysis of SMC

**Fig. 12:**
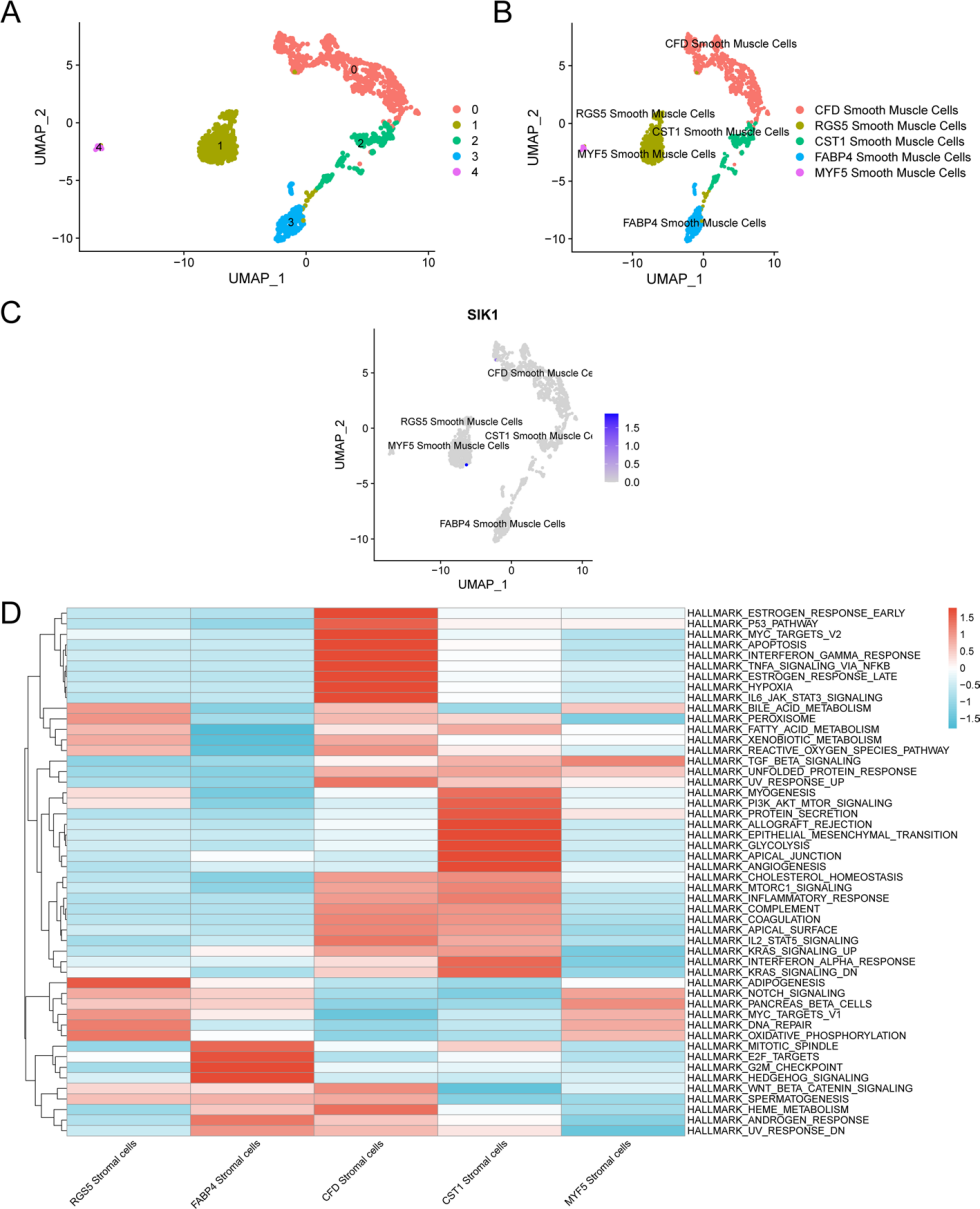
Sub type analysis of SMC. **A** UMAP map of cell clustering of SMC. **B** SMC, Cells after annotation UMAP figure. **C** UMAP diagram of the expression of Model gene SIK1 in Smooth Muscle cell subsets. **D** Heatmap of GSVA among Smooth Muscle cell subsets

To explore new subsets of SMC, we extracted the SMC cluster from the GSE184362 dataset for clustering. Five SMC subsets (Fig. 12A) were obtained. Subsequently, we employed the “FindAllMarkers” function to identify differentially expressed genes between the two subsets, annotating them with the significant DEGs as shown in Fig. 12B. This process ultimately resulted in the identification of *CFD SMC. RGS5 SMC*,* CST1 SMC*,* FABP4 SMC*,* MYF5 SMC*. To assess the differences in expression levels of the two Model Genes across the SMC subsets, we utilized a UMAP diagram to visualize their expression patterns among these subsets (Fig. 12C). The findings indicated that the expression of the Model Gene *SIK1* was notably higher in RGS5 SMC compared to the other SMC subsets.

To investigate the differences among the 50 gene sets from H. all.v7.4.Symbols.gmt across the five SMC subsets, we conducted GSVA on all genes and subsequently created a heatmap for visualization (Fig. 12D).

## Discussion

THCA is among the most prevalent endocrine cancers, significantly impacting patients’ health and quality of life. This malignancy encompasses several subtypes, including papillary, follicular, medullary, and anaplastic THCA, each exhibiting unique biological characteristics and clinical outcomes. The most common subtype, PTC, is noted for its relatively favorable prognosis. In contrast, ATC is characterized by its high aggressiveness and is associated with poor survival rates [34]. The rising incidence of THCA, especially in iodine-deficient regions, highlights the urgent need for effective diagnostic and therapeutic approaches to manage this disease [35]. Gaining insights into the molecular mechanisms that drive thyroid carcinoma progression and confer treatment resistance is essential for enhancing patient prognosis and outcomes.

Ubiquitination, a post-translational modification process, plays a vital role in regulating protein stability and function, thereby influencing various cellular processes, including cell cycle, apoptosis, and DNA repair. Ubiquitination is critically involved in the development and progression of thyroid cancer through multiple mechanisms. Proteins destined for degradation are marked through the ubiquitin-proteasome pathway, a critical mechanism that sustains cellular homeostasis by eliminating dysfunctional proteins. In the context of thyroid cancer, the ubiquitination and degradation of tumor suppressor proteins such as p53 and Rb promote tumor cell growth and survival, indicating that dysregulation of this pathway facilitates tumorigenesis. Cancer cells evade immune surveillance by modulating the expression of antigen-presenting and immune-suppressive factors, with E3 ubiquitin ligases enhancing immune evasion capabilities through the regulation of immune checkpoint molecules. Furthermore, with respect to treatment involving radioactive iodine, ubiquitination has been associated with therapeutic resistance, attributed to the abnormal expression of certain URGs in RAI-refractory cells [36]. For instance, the ubiquitin-proteasome system (UPS) components have been found to be differentially expressed in RAI-refractory thyroid cancer cells, suggesting their potential role in modulating treatment response [37]. Targeting ubiquitination-related pathways presents significant potential for drug development, as these therapies may restore tumor suppressor protein expression, enhance immune responses, and improve treatment sensitivity. Overall, understanding the role of ubiquitination in protein degradation, signal transduction, immune evasion, and therapeutic resistance is essential for advancing therapeutic strategies in thyroid cancer.

Our study identified 25 URDEGs in THCA that are potentially relevant to RAI therapy response. GO and KEGG enrichment analyses revealed significant involvement of these genes in biological processes such as cytoskeleton organization and digestive tract development, and pathways including the PPAR signaling pathway.

The PPAR signaling pathway, particularly PPAR-γ, plays a vital role in the biology of thyroid cancer. For example, the PAX8-PPAR-γ fusion oncogene found in follicular thyroid carcinomas (FTCs) generates a unique transcriptional profile that influences cell growth and various signal transduction pathways [38]. Additionally, PPAR-γ agonists like Lobeglitazone have been demonstrated to inhibit EMT and metastatic behaviors in PTC by suppressing the p38 MAPK signaling pathway [39]. This suggests that PPAR-γ modulation could be a therapeutic target for managing THCA, particularly in RAI-refractory cases.

Moreover, our analysis identified pathways such as the MAPK signaling pathway and proteoglycans in cancer, which have been implicated in thyroid cancer progression and treatment resistance. The MAPK pathway, frequently activated by mutations in genes such as BRAF, is a well-established contributor to the proliferation and survival of thyroid cancer cells [40]. Inhibiting this pathway has shown promise in reducing tumor growth and enhancing RAI uptake.

Comparing our findings with previous studies, we observed that the identified URDEGs and their associated pathways align with known molecular mechanisms in thyroid cancer. For instance, the overexpression of PPAR-δ, which induces cell proliferation through cyclin E1, has been reported in various thyroid tumors, highlighting its potential as a therapeutic target [41]. Additionally, the role of 15-deoxy-Δ(12,14)-prostaglandin J2 in inducing apoptosis and upregulating *SOCS3* in thyroid cancer cells further supports the therapeutic relevance of targeting PPAR signaling [42].

In conclusion, our study highlights the importance of ubiquitination-related genes and their involvement in key signaling pathways such as PPAR, MAPK, and proteoglycans. These results establish a foundation for the development of targeted therapies aimed at increasing RAI sensitivity and addressing resistance in THCA.

In our investigation of the immune characteristics and mutation profiles of THCA, we identified significant insights that align with and extend existing research. Our study revealed that T-cell helper cells, monocytes, M1 macrophages, and resting dendritic cells exhibited significant differences between high and low-risk groups. These findings are consistent with previous studies indicating that immune cell infiltration plays a crucial role in the tumor microenvironment of thyroid cancer. For instance, a study by Means et al. demonstrated that distinct immune microenvironments in PTC correlate with pathological aggressiveness, particularly noting higher CD8+ T cells in less aggressive tumors and increased mast cell density associated with BRAF mutations [43]. This suggests that immune cell composition can be indicative of tumor behavior and patient prognosis.

Furthermore, the tumor mutation burden (TMB) in THCA has been shown to be a critical factor in prognosis. Xie et al. reported that high TMB in PTC is associated with poorer progression-free survival and is characterized by lower immune cell infiltration and a higher proportion of tumor-promoting immune cells [44]. This aligns with our findings, where high TMB correlated with more aggressive disease features and reduced immune response.

Comparatively, our study identified specific URDEGs such as *INSM2*, *TAGLN3*, *MDGA2*, and *SIK1*, which were incorporated into a prognostic risk model. These genes have shown significant associations with prognosis, similar to the findings of Ji et al., who highlighted the prognostic value of m6A methylation modifications and their impact on immune cell infiltration in thyroid cancer [45]. This further underscores the potential of specific gene signatures in predicting patient outcomes and guiding treatment strategies.

When evaluating the efficacy of our URDEGs as biomarkers for immune therapy response, they demonstrated promising potential. This finding is consistent with the research conducted by Zhou et al., who explored the prognostic and immunotherapeutic value of *CD274* and *PDCD1LG2* across various cancers, highlighting their role in predicting immune response and patient survival [46]. Our URDEGs, when compared to these established markers, showed comparable predictive performance, suggesting their utility in clinical settings.

In summary, our study adds to the growing evidence that underscores the complex interplay between immune characteristics, mutation profiles, and prognosis in THCA. The identified URDEGs not only serve as potential biomarkers for prognosis but also offer insights into the underlying mechanisms of immune evasion and tumor progression, providing a foundation for future therapeutic strategies.

Our study identified several ubiquitination-related genes as potential therapeutic targets for enhancing the efficacy of RAI therapy in THCA. Among the notable findings, the E3 ubiquitin ligases *FBXL19* and *HECW1* were found to regulate the degradation of thyroid transcription factor 1 (*TTF1)* in thyroid carcinoma cells, suggesting their role in modulating thyroid cancer cell behavior and response to RAI therapy [47]. Additionally, the *COP9* signalosome subunit 5 (*CSN5*) was shown to promote thyroid carcinoma progression by stabilizing angiopoietin-like protein 2 (*ANGPTL2*), implicating *CSN5* as having a role in thyroid cancer that warrants further investigation and may have potential therapeutic significance [48]. Another significant finding was the downregulation of the transmembrane E3 ubiquitin ligase *ZNRF3* in PTC, which negatively regulates β-catenin activation and suppresses tumor progression, highlighting its potential as a tumor suppressor [49].

Our findings are consistent with earlier research that highlights the critical role of ubiquitination in thyroid cancer. Specifically, the overexpression of S-phase kinase-associated protein 2 (Skp2) in thyroid cancer has been associated with enhanced degradation of p27, contributing to tumor progression. This supports the potential for therapeutic strategies targeting Skp2 in THCA [50]. Moreover, the ubiquitin-proteasome pathway has been linked to the regulation of cell proliferation and survival across various cancers, including THCA. This occurs through the modulation of essential signaling pathways such as NF-κB and p53 [51].

The identification of these URDEGs opens new avenues for targeted therapy in THCA. Future investigations should prioritize the validation of these targets in preclinical models, as well as examine their potential when combined with current therapeutic approaches, such as RAI and chemotherapy. Additionally, the development of specific inhibitors or modulators of these ubiquitination-related proteins could provide novel therapeutic strategies for improving the outcomes of patients with THCA.

In reflecting on the limitations of this study, several aspects warrant consideration. Firstly, the research primarily relies on bioinformatics analyses and has not integrated experimental validation through wet lab techniques, such as IHC or qPCR, which could provide additional validation and deeper mechanistic insights into the identified biomarkers. While the study utilizes large datasets from reputable sources such as TCGA and GEO, the sample size may still be limited in capturing the full heterogeneity of thyroid cancer (THCA), potentially affecting the generalizability of the findings. Furthermore, the study lacks clinical validation, and thus, the prognostic risk model and identified biomarkers have not yet been tested in clinical settings, which is crucial for assessing their practical applicability in a real-world context. Another consideration is that the use of multiple datasets introduces the possibility of batch effects, which, despite efforts in standardization, might still influence the results. Lastly, it is important to note that this study is exploratory in nature, and the findings are primarily hypothesis-generating; as such, the biological roles of the identified genes in thyroid cancer differentiation and iodine uptake will require further validation through in vitro and in vivo experiments. These limitations have been addressed in the revised manuscript, where we emphasize the exploratory scope of the research and outline future directions for experimental validation.

## Conclusion

In summary, this study successfully identified URDEGs associated with RAI therapy sensitivity and resistance in THCA. Through comprehensive bioinformatics analyses, including differential expression, enrichment, and single-cell RNA sequencing analyses, we constructed a prognostic risk model with potential clinical utility. These findings enhance our understanding of the molecular mechanisms that influence the response to RAI therapy, opening up new possibilities for therapeutic interventions. Future studies should focus on validating these findings through experimental research and clinical trials, with the goal of improving the precision of treatment strategies for THCA.

## Supplementary Information

Below is the link to the electronic supplementary material.

Supplementary Material 1.

Supplementary Material 2.

Supplementary Material 3.

Supplementary Material 4.

Supplementary Material 5.

## Data Availability

The datasets generated and/or analysed during the current study are available in the Repository: Cancer Genome Atlas database (https://portal.gdc.cancer.gov/). UCSC Xena database (https://xena.ucsc.edu/). GEO database (https://www.ncbi.nlm.nih.gov/geo/). GeneCards database (https://www.genecards.org/).

## Abbreviations

THCA: Thyroid carcinoma
URDEGs: Ubiquitination-related differentially expressed genes
TCGA: The Cancer Genome Atlas
GEO: Gene Expression Omnibus
GO: Gene Ontology
KEGG: Kyoto Encyclopedia of Genes and Genomes
LASSO: Absolute Shrinkage and Selection Operator
GSVA: Gene Set Variation Analysis
PPAR: Proliferator-activated receptor
RAI: Radioactive iodine
ICIs: Immune checkpoint inhibitors
irAEs: Immune-related adverse events
TCGA-THCA: Thyroid cancer data set
RAI Resistance PTC: Radioactive iodine resistance papillary thyroid cancer
RAI After PTC: Radioactive iodine after papillary thyroid cancer
RAI Before PTC: Radioactive iodine before papillary thyroid cancer
URGs: Ubiquitin-related genes
BH method: Benjamini–Hochberg method
BP: Biological processes
CC: Cellular components
MF: Molecular functions
ROC: Receiver operating characteristic
PFI: Progression-free interval
AUC: Area under the curve
KM curve: Kaplan-Meier curve
PCA: Principal component analysis
UMAP: Uniform manifold approximation and projection
scDEGs: Single-cell group differentially expressed genes
PTC: Papillary thyroid carcinoma
MG: Model genes
DCs: Dendritic cells
EC: Endothelial cells
SMC: Smooth muscle cells
TMB: Tumor mutation burden
TTF1: Thyroid transcription factor 1
CSN5: COP9 signalosome subunit 5
ANGPTL2: Angiopoietin-like protein 2
Skp2: S-phase kinase-associated protein 2
