# Supplementary figures and images for: Exploring molecular mechanisms of radioactive iodine therapy in thyroid cancer using single-cell RNA sequencing data
Source: Discov Oncol. 2026 Jan 3;17:213. doi: 10.1007/s12672-025-04317-x (PMC12864557; doi:10.1007/s12672-025-04317-x)

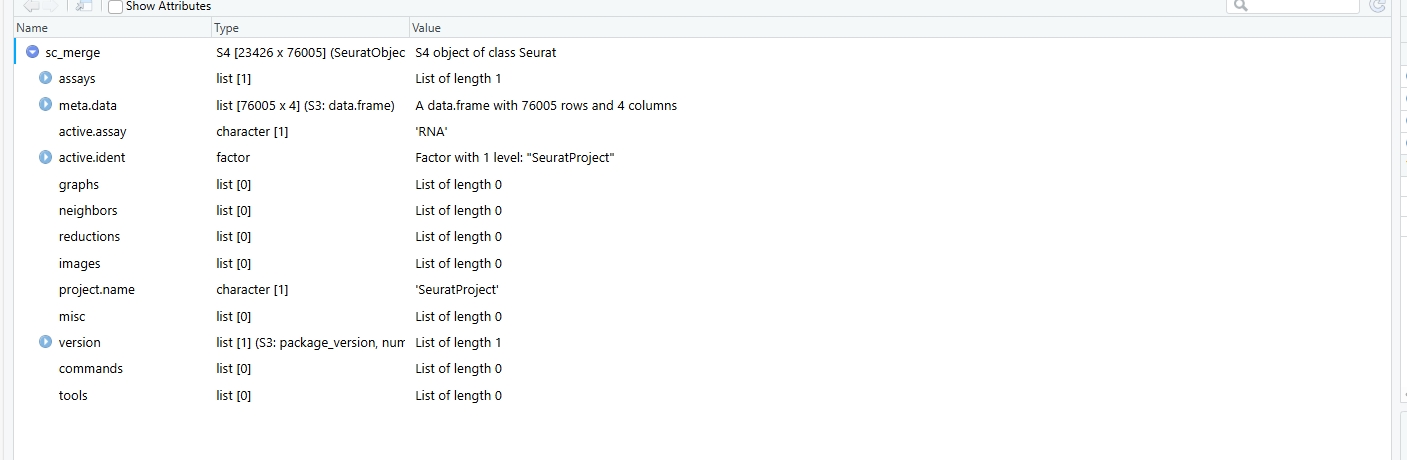

Supplement: Supplementary file 4 — Supplementary Material 4. [file 12672_2025_4317_MOESM4_ESM.jpg]

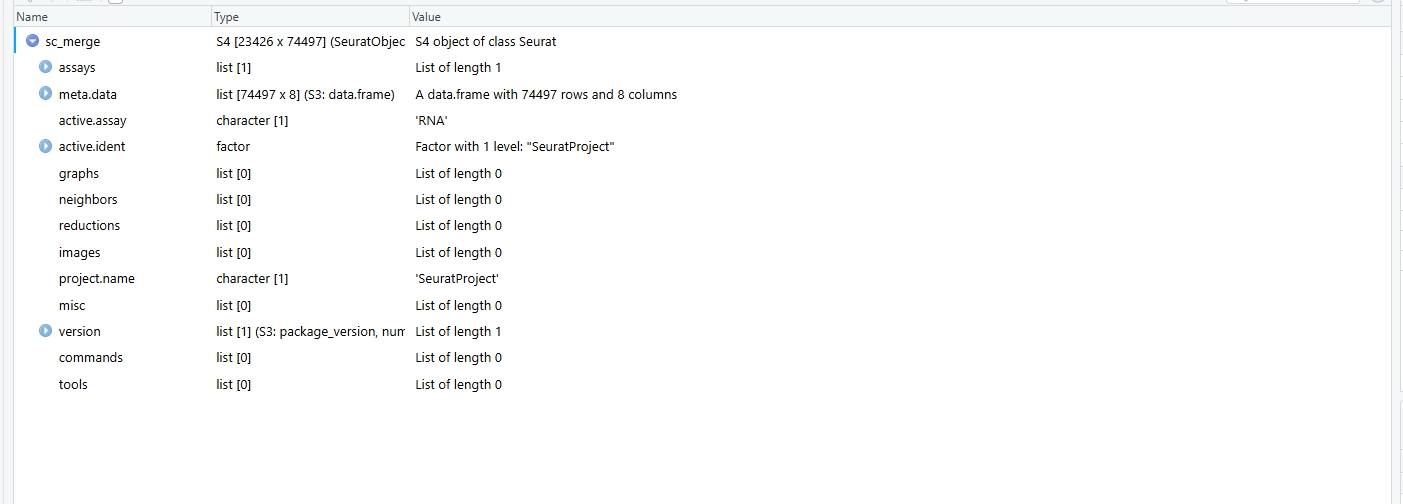

Supplement: Supplementary file 5 — Supplementary Material 5. [file 12672_2025_4317_MOESM5_ESM.jpg]
